# Supplementary material for: Structural insights into the cross-exon to cross-intron spliceosome switch
Source: Nature. 2024 May 22;630(8018):1012–9. doi: 10.1038/s41586-024-07458-1 (PMC11208138; doi:10.1038/s41586-024-07458-1)
Supplement: Supplementary file 1 — Source data for RNA gels and western blots. a–d, Source (uncropped) gels used to generate the figures showing the RNA composition of the various spliceosomal complexes that were analysed by cryo-EM. e,f Source (uncropped) western blots used to generate Extended Data Fig. 7f. The same western blot was first immunostained with antibodies against phosphorylated human PRP6 and phosphorylated human PRP31, then stripped and subsequently immunostained with antibodies against human SF3B1 (a loading control), PRP6 and PRP31. Dotted boxes indicate the regions of each gel/blot shown in the indicated Extended Data figures, as indicated below. [file 41586_2024_7458_MOESM1_ESM.pdf]

nature portfolio

https://doi.org/10.1038/s41586-024-07458-1

Supplementary information

Structural insights into the cross-exon to cross-intron spliceosome switch

In the format provided by the authors and unedited

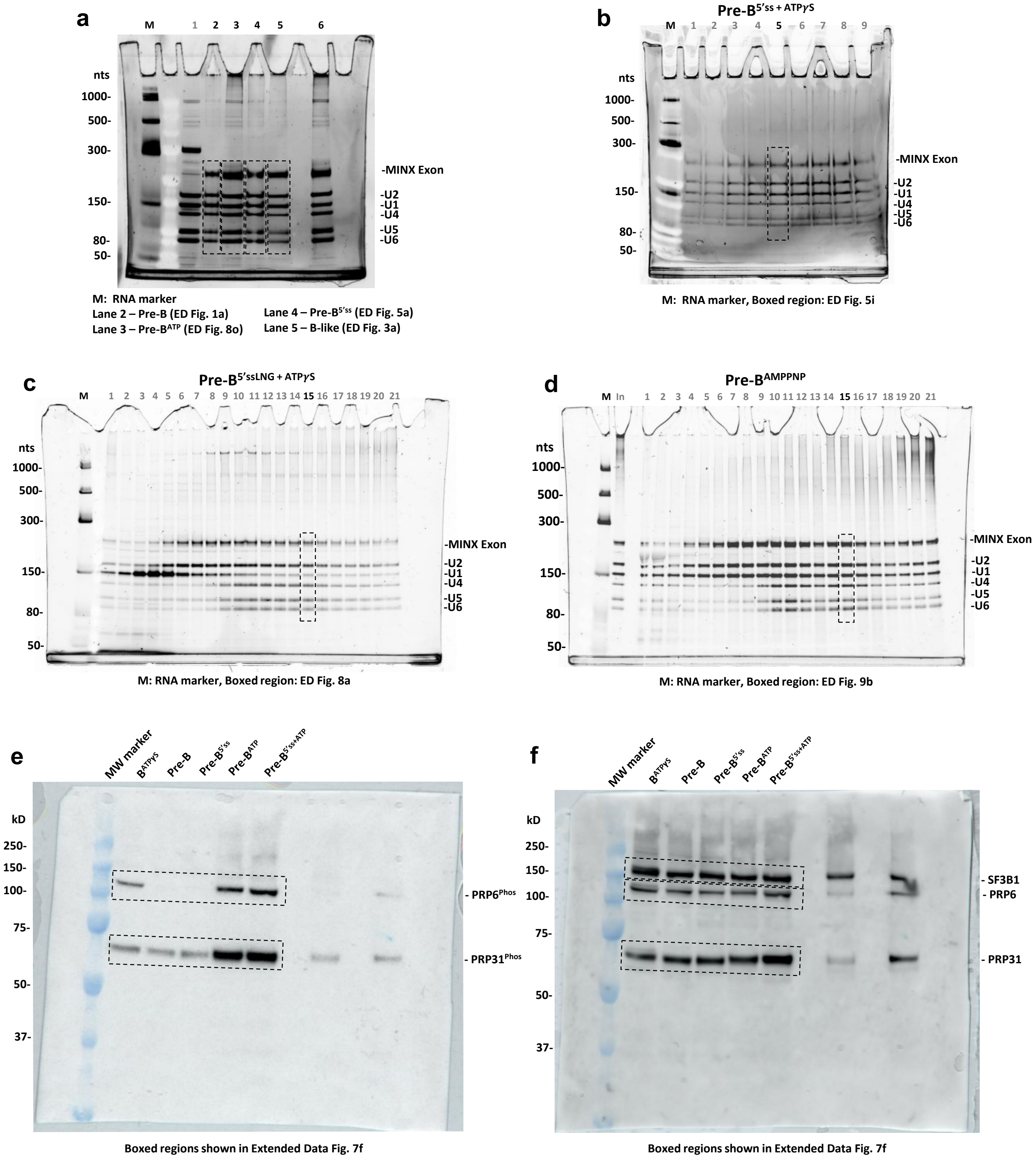

Supplementary Figure 1. Source data for RNA gels and Western blots. a-d, Source (uncropped) gels used to generate the figures showing the RNA composition of the various spliceosomal complexes that were analysed by cryo-EM. **e,f** Source (uncropped) western blots used to generate Extended Data Fig. 7f. The same Western blot was first immunostained with antibodies against phosphorylated human PRP6 and phosphorylated human PRP31, then stripped and subsequently immunostained with antibodies against human SF3B1 (a loading control), PRP6 and PRP31. Dotted boxes indicate the regions of each gel/blot shown in the indicated Extended Data figures, as indicated below.
