# Supplementary material for: Structural insights into the cross-exon to cross-intron spliceosome switch
Source: Nature. 2024 May 22;630(8018):1012–9. doi: 10.1038/s41586-024-07458-1 (PMC11208138; doi:10.1038/s41586-024-07458-1)
Supplement: Supplementary file 3 — Protein composition of CE pre-B and B-like complexes. Proteins were identified by searching uHPLC-ESI MS raw data with MaxQuant at a false discovery rate (FDR) of 1% (at both peptide spectrum match (PSM) and protein levels). Only the 100 most abundant proteins detected in either complex as judged by intensity based absolute quantitation (iBAQ) values are shown. The asterisk marks the recombinant protein used for affinity purification that is present in 6 copies per complex. Contaminants commonly found in MS samples were excluded from the table. [file 41586_2024_7458_MOESM3_ESM.pdf]

Supplementary Table 1. Protein composition of cross-exon pre-B and B-like complexes. Proteins were identified by searching uHPLC-ESI MS raw data with MaxQuant at false discovery rate 1% (at both peptide spectrum match (PSM) and protein levels). Only the 100 most-abundant proteins detected in either complex as judged by intensity based absolute quantitation (iBAQ) values are shown. Asterisk marks a recombinant protein used for affinity purification that is present in 6 copies per complex. Contaminants commonly found in MS samples were excluded from the table.

			**CE pre-B dimer**			**CE B-like dimer**		
**Name**	**Uniprot AC**	**kDa**	**PSMs**	**iBAQ**	**iBAQ RANK**	**PSMs**	**iBAQ**	**iBAQ RANK**
**Sm proteins**								
SMB/B'	P14678	24.6	202	1.27E+09	**49**	275	2.05E+09	**22**
SMD1	P62314	13.3	56	6.40E+08	**78**	135	5.31E+09	**5**
SMD2	P62316	13.5	197	7.99E+09	**2**	302	8.00E+09	**4**
SMD3	P62318	13.9	143	2.31E+09	**20**	361	4.32E+09	**7**
SME	P62304	10.8	86	2.13E+09	**23**	260	8.98E+09	**3**
SMF	P62306	9.7	77	1.51E+09	**41**	102	3.54E+09	**9**
SMG	P62308	8.5	32	3.29E+09	**7**	110	9.51E+09	**2**
**U1 snRNP**								
U1-70K	P08621	51.6	256	2.90E+09	**12**	246	3.30E+09	**11**
U1-A	P09012	31.3	209	1.38E+09	**44**	150	1.52E+09	**37**
U1-C	P09234	17.4	83	5.87E+08	**84**	52	9.99E+08	**53**
**U1-related**								
Luc7-like 2	Q9Y383	46.5	141	8.95E+08	**62**	93	5.06E+08	**73**
Luc7-like 3	O95232	51.5	119	7.82E+08	**69**	145	7.75E+08	**60**
PRPF40A	O75400	108.8	342	6.92E+08	**74**	210	3.73E+08	**81**
RBM25	P49756	100.2	386	6.16E+08	**81**	218	3.69E+08	**82**
RBM39	Q14498	58.5	429	2.48E+09	**16**	311	2.37E+09	**19**
**U2 snRNP**								
U2A'	P09661	28.4	179	3.08E+09	**9**	162	2.18E+09	**21**
U2B"	P08579	25.5	137	1.38E+09	**45**	165	1.18E+09	**48**
SF3A1	Q15459	88.9	598	1.26E+09	**50**	430	1.11E+09	**51**
SF3A2	Q15428	49.3	207	8.50E+08	**63**	181	9.88E+08	**54**
SF3A3	Q12874	58.9	387	1.86E+09	**28**	361	1.63E+09	**36**
SF3B1	O75533	145.8	1,199	1.75E+09	**33**	860	1.67E+09	**33**
SF3B2	Q13435	100.2	876	1.59E+09	**36**	653	1.21E+09	**45**
SF3B3	Q15393	135.6	1,080	3.12E+09	**8**	807	2.48E+09	**18**
SF3B4	Q15427	44.4	96	6.34E+08	**79**	171	6.99E+08	**64**
SF3B6	Q9Y3B4	14.6	91	2.28E+09	**21**	62	1.83E+09	**27**
PHF5A	Q7RTV0	12.4	76	2.81E+09	**13**	62	2.67E+09	**15**
SF3B5	Q9BWJ5	10.1	126	2.76E+09	**14**	124	3.75E+09	**8**
**U2-related**								
DDX42	Q86XP3	103	203	1.04E+08	**161**	273	3.38E+08	**84**
DHX15 (PRP43)	O43143	90.9	466	8.09E+08	**66**	292	6.37E+08	**69**
SPF30	O75940	26.7	95	1.22E+09	**52**	116	1.41E+09	**42**
PUF60	Q9UHX1	59.9	346	1.35E+09	**46**	240	1.34E+09	**43**
U2AF35	Q01081	27.9	153	1.85E+09	**30**	140	2.54E+09	**16**
U2AF65	P26368	53.5	437	1.44E+09	**43**	369	1.75E+09	**31**
**U5**								

			CE pre-B dimer			CE B-like dimer		
**Name**	**Uniprot AC**	**kDa**	**PSMs**	**iBAQ**	**iBAQ RANK**	**PSMs**	**iBAQ**	**iBAQ RANK**
PRP8	Q6P2Q9	273.6	2,283	2.15E+09	**22**	1,524	1.64E+09	**35**
BRR2	O75643	244.5	2,449	2.41E+09	**18**	1,676	1.81E+09	**29**
SNU114	Q15029	109.4	1,157	2.99E+09	**10**	709	2.71E+09	**14**
U5-40K	Q96DI7	39.3	329	1.20E+09	**53**	281	1.44E+09	**41**
PRP6	O94906	106.9	657	1.77E+09	**32**	505	1.49E+09	**39**
DIM1	P83876	16.8	202	8.98E+08	**61**	163	1.19E+09	**47**
PRP28	Q9BUQ8	95.6	715	2.09E+09	**24**	428	1.19E+09	**46**
**LSm proteins**								
LSm2	Q9Y333	10.8	84	1.15E+09	**55**	80	1.94E+09	**24**
LSm3	P62310	11.8	57	2.32E+09	**19**	87	3.42E+09	**10**
LSm4	Q9Y4Z0	15.4	119	6.66E+08	**75**	82	7.21E+08	**63**
LSm5	Q9Y4Y9	9.9	46	1.28E+09	**48**	40	1.69E+09	**32**
LSm6	P62312	9.1	42	2.01E+09	**26**	26	1.30E+09	**44**
LSm7	Q9UK45	11.6	28	2.67E+08	**105**	69	5.58E+08	**72**
LSm8	O95777	10.4	59	1.77E+09	**31**	126	2.50E+09	**17**
**U4/U6**								
PRP3	O43395	77.6	568	1.25E+09	**51**	426	1.06E+09	**52**
PRP4	O43172	58.5	486	1.69E+09	**34**	358	1.77E+09	**30**
PPIH	O43447	19.2	143	9.26E+08	**60**	101	7.51E+08	**61**
PRP31	Q8WWY3	55.5	397	1.86E+09	**29**	258	1.65E+09	**34**
SNU13	P55769	14.2	80	1.56E+09	**37**	59	1.85E+09	**25**
**U4/U6.U5**								
SNU66	O43290	90.3	700	1.94E+09	**27**	489	1.96E+09	**23**
SAD1	Q53GS9	65.4	339	2.03E+09	**25**	301	1.82E+09	**28**
SNRNP27	Q8WVK2	18.9	96	1.66E+09	**35**	48	1.16E+09	**49**
RBM42	Q9BTD8	50.4	209	1.53E+09	**40**	123	9.08E+08	**58**
PRP4 kinase	Q13523	117	587	9.52E+08	**59**	341	6.47E+08	**67**
**B-specific proteins**								
SNU23 (ZMAT2)	Q96NC0	23.6	27	3.20E+07	**238**	52	2.21E+08	**96**
PRP38A	Q8NAV1	37.5	116	4.98E+08	**91**	99	5.02E+08	**74**
SMU1 (fSAP57)	Q2TAY7	57.5	311	8.22E+08	**65**	244	9.77E+08	**55**
MFAP1	P55081	52	89	1.90E+08	**122**	76	2.76E+08	**86**
RED	Q13123	65.6	214	4.85E+08	**92**	167	4.55E+08	**78**
FBP21	O75554	42.5	36	3.73E+07	**229**	83	2.27E+08	**95**
UBL5	Q9BZL1	8.5	17	9.06E+07	**175**	24	4.83E+08	**76**
**Cap binding complex**								
NCBP2	P52298	18	47	5.49E+08	**87**	57	6.10E+08	**70**
NCBP1	Q09161	91.8	479	8.28E+08	**64**	508	9.12E+08	**57**
**SR proteins**								
SRSF1	Q07955	27.8	230	5.85E+09	**4**	200	4.42E+09	**6**
SRSF7	Q16629	27.4	114	4.02E+09	**5**	80	2.91E+09	**13**
SRSF3	P84103	19.3	53	1.55E+09	**38**	42	6.44E+08	**68**
SRSF5	Q13243	31.3	50	2.13E+08	**115**	41	2.44E+08	**91**
SRSF6	Q13247	39.6	70	7.62E+08	**71**	46	4.32E+08	**79**
SRSF2	Q01130	25.5	170	2.53E+09	**15**	116	2.26E+09	**20**
SFRS12	Q8WXA9	59.4	40	1.36E+08	**144**	53	2.52E+08	**90**

			CE pre-B dimer			CE B-like dimer		
**Name**	**Uniprot AC**	**kDa**	**PSMs**	**iBAQ**	**iBAQ RANK**	**PSMs**	**iBAQ**	**iBAQ RANK**
SRS11	Q05519	53.5	117	6.48E+08	**77**	103	6.86E+08	**65**
TRA2B	P62995	33.7	73	6.08E+08	**83**	24	1.62E+08	**109**
**SR-related proteins**								
FLJ10154	Q9NWB6	33.2	37	8.05E+08	**67**	26	2.35E+08	**94**
**hnRNP**								
HNRNP A0	Q13151	30.9	119	1.19E+09	**54**	129	1.15E+09	**50**
HNRNP A1	P09651	38.7	432	2.46E+09	**17**	310	2.91E+09	**12**
HNRNP A3	P51991	39.6	229	6.58E+08	**76**	75	1.54E+08	**110**
HNRNP A2/B1	P22626	37.4	404	2.92E+09	**11**	192	9.49E+08	**56**
HNRNP C	P07910	33.7	180	1.32E+09	**47**	21	4.48E+07	**151**
HNRNP H1	P31943	49.2	178	5.23E+08	**88**	76	1.83E+08	**101**
HNRNP L	P14866	64.1	192	4.26E+08	**94**	37	2.26E+07	**196**
PCBP1	Q15365	37.5	162	6.10E+08	**82**	105	8.09E+08	**59**
**Other factors**								
MSI2	Q96DH6	35.2	90	6.30E+08	**80**	62	4.65E+08	**77**
PRP38B	Q5VTL8	64.5	118	2.12E+08	**116**	109	2.41E+08	**93**
ERH	P84090	12.3	93	3.84E+09	**6**	37	7.37E+08	**62**
MGN2	Q96A72	17.3	91	3.66E+08	**98**	3	3.98E+06	**310**
RSRC2	Q7L4I2	50.6	117	7.68E+08	**70**	89	6.76E+08	**66**
TOE1	Q96GM8	56.5	72	9.22E+07	**172**	139	4.22E+08	**80**
EIF4A3	P38919	46.9	254	1.11E+09	**56**	23	7.50E+06	**264**
MAGOH	Q96A72	17.2	91	3.66E+08	**98**	3	3.98E+06	**310**
RBM8A (Y14)	Q9Y5S9	19.9	128	7.48E+08	**72**	5	3.21E+06	**327**
PININ	Q9H307	81.6	204	3.51E+08	**99**	59	3.68E+07	**165**
RNPS1	Q15287	34.2	152	5.73E+08	**85**	32	1.41E+08	**111**
SAP18	O00422	17.6	94	1.01E+09	**58**	36	1.72E+08	**104**
THOC4 (ALY/REF)	Q86V81	26.9	137	5.67E+08	**86**	18	4.53E+07	**150**
UAP56	Q13838	49	224	7.87E+08	**68**	42	3.18E+07	**172**
PABPN1, PAB2	Q86U42	32.8	75	5.20E+08	**89**	2	3.92E+06	**311**
SRRM2	Q9UQ35	300	600	4.19E+08	**95**	289	2.56E+08	**89**
BUB3	O43684	37.2	177	1.47E+09	**42**	192	1.84E+09	**26**
ZNF207	O43670	50.8	99	5.08E+08	**90**	72	4.93E+08	**75**
SF4 (F23858)	Q8IWZ8	72.5	235	2.60E+08	**107**	182	2.44E+08	**92**
PRP19	Q9UMS4	55.2	302	1.04E+09	**57**	130	2.68E+08	**88**
SPF27	O75934	26	94	3.78E+08	**96**	42	1.27E+08	**117**
FUS	P35637	53.4	108	4.66E+08	**93**	95	5.74E+08	**71**
PPP1R8 (NIPP1)	Q12972	38.5	85	9.05E+07	**176**	84	2.14E+08	**97**
ZC3H18 (NHN1)	Q86VM9	106.4	294	3.66E+08	**97**	219	2.69E+08	**87**
SRRT (ASR2B)	Q9BXP5	100	598	1.54E+09	**39**	487	1.45E+09	**40**
DDX5 (p68)	P17844	69.2	309	6.94E+08	**73**	127	2.13E+08	**98**
AGGF1	Q8N302	80.977	92	9.19E+07	**173**	155	1.97E+08	**99**
BUD31	P41223	17.0	50	2.00E+8	**118**	34	1.84E+08	**100**
MBP-MS2*		56.9	1,338	1.32E+10	**1**	1,287	1.16E+10	**1**
